# Towards Sustainable Cities: The Spillover Effects of Waste-Sorting Policies on Sustainable Consumption

**DOI:** 10.3390/ijerph182010975

**Published:** 2021-10-19

**Authors:** Shu Yang, Peng Cheng, Shanyong Wang, Jun Li

**Affiliations:** 1 College of Economics and Management, China Agricultural University, Beijing 100083, China; yangshuxy@126.com; 2 Beijing Food Safety Policy & Strategy Research Base, Beijing 100083, China; 3 Department of Marketing & Logistics Management, Nanjing University of Finance and Economics, Nanjing 210046, China; 4 Department of Public Affairs, University of Science and Technology of China, Hefei 230026, China; wsy1988@ustc.edu.cn; 5 School of Economics, Hefei University of Technology, Hefei 230009, China; economics-lijun@hfut.edu.cn

**Keywords:** waste sorting, sustainable consumption, regulatory policy, spillover effect

## Abstract

The increasing amount of waste in cities poses a great challenge for sustainable development. Promoting waste sorting is one of the priorities for various levels of public authorities in the context of the rapid growth of waste generation all around China. To achieve this goal, waste-sorting policies should be precisely designed to ensure successful waste reduction at all stages. Previous studies have neglected the spillover effects of different regulatory policies, which may affect the overall goal of reducing waste by influencing different waste production stages. This paper fills this gap by comparing the spillover effects of two typical waste-sorting policies on sustainable consumption behaviours through a survey conducted in Shanghai and Beijing (control group). By combining quasi-natural experiment and questionnaire methods, this paper analyses data through a mediation test to explore the spillover effects between different regulatory policy groups and the effects of the mediation psychological factors. Results show that a penalty policy significantly decreases people’s sustainable consumption behaviours through a negative spillover effect, while a voluntary participation policy significantly increases sustainable consumption behaviours through a positive spillover effect. Results can provide implications for policymaking in waste management and other pro-environmental fields to help cities become more sustainable by shifting multiple behaviours.

## 1. Introduction

With the recent increasing severity of ‘garbage sieges’ [1] in Chinese cities, the policy of separating household waste for disposal has been raised to an unprecedented level. The National Development and Reform Commission and the Ministry of Housing and Urban–Rural Development jointly issued their Mandatory Waste Sorting System Program (Draft) in June 2016. To promote waste-sorting behaviour, the Chinese government then chose several cities as pilot areas for garbage classification. Respectively, these cities designed various regulatory policies aimed at transforming human behaviour towards improved waste sorting and minimisation.

For these cities, waste-sorting policies should be precisely designed to ensure successful waste reduction at all stages to achieve the waste reduction goal. Not only should waste sorting be promoted, the amount of waste produced upstream also deserves attention. Hence both waste-sorting behaviour and upstream consumption and waste production stand out as crucial stages of reaching the overall environmental goal.

Does the downstream waste-sorting policy design affect the upstream sustainable consumption? It is an intriguing and vital question because the answer relies on the same environmental goal of waste reduction. Additionally, the comprehensive evaluation of behaviours is a trend in current pro-environmental studies and policy areas [2]. The spillover theory in pro-environmental behaviour research has shed light on this problem and provides an alternative study path for this indirect impact. The spillover effect refers to past pro-environment behaviour (PEB), which affects the possibility or depth of participation of the subject in other PEBs in the future or the positive or negative impact of regulatory policies on non-target PEBs [3,4]. Most of the current studies consider the first concept in evaluating an initial behaviour’s impact on subsequent ones. Although an emerging body of research is focusing on the second concept, most studies conduct laboratory experiments to verify different information framings’ effects rather than the effects of different types of policies [5].

From the perspective of policymakers, research indicates that the interventions targeted at initial behaviours can either trigger environmentally friendly behaviours or decrease other PEBs through positive or negative spillover effects [6]. Whether the influence is positive or negative has a specific relationship with the type of intervention [7,8]. As classified by [9], pro-environmental interventions include any attempt to encourage behaviour change, i.e., ‘a request to perform a new behaviour, public education campaign, tax incentive, provision of green infrastructure such as kerbside recycling, and regulatory policy’. As a typical behaviour with externality, regulatory policies are widely used to promote people’s behavioural transformation. Investigating the spillover effects of different policies so as to select a policy with high overall environmental benefits is of great significance. Such research facilitates the realisation of more pro-environmental behavioural changes through ingenious policy design targeted at triggering out positive spillovers. However, most research has focused on information framings’ effects, and few studies have explored the spillover effects of regulatory policy, let alone conducted the effect comparison of different types of policy in the real context. Xu et al. [10] examined the environmental and incentive policies’ spillover effects in Hangzhou but found no difference between these two policies. This result is unusual relative to the past spillover effects using information framings. Furthermore, the effects of different environmental behaviour-promoting policies have seldom been studied before, such as penalty and incentive policies’ spillover effects.

How different regulatory policies influence multiple pro-environmental behaviours directly affects the overall effects of the policies on the environment. To that end, this paper aims to distinguish the effects of different policies regarding waste sorting and specify the type of policy that can promote sustainable consumption behaviours. We investigate the spillover effects of various regulatory policies targeted at the initial waste-sorting behaviours on the subsequent sustainable consumption ones. The psychological mechanisms mediating the spillover effect are also explored. By solving these problems, this study reveals different regulatory policies’ spillover effects to evaluate the overall environmental influence and explains the potential psychological mechanism to shed light on the policy design and process intervention. This paper provides both theoretical and practical contributions. Firstly, it fills a gap and enriches the current spillover effect studies in the PEB realm by examining different initial regulatory policies’ spillover effects rather than initial behaviours or information framings. This work demonstrates that different policies elicit different spillover effects onto other PEBs. As Xu et al. [10] indicated, current works on the effects of regulatory policies on spillover effects are limited; thus, such an empirical paper is essential. It verifies different regulatory policies’ spillover effects and adds to the knowledge from the policy side. Secondly, waste-sorting and sustainable consumption behaviours are highly correlated to daily residential behaviours, and the latter generate a considerable portion of household carbon emissions [11,12,13]. The spillover effects of waste-sorting policies are evaluated through a combination of two behaviours, a targeted behaviour and a non-targeted one. The results provide policymakers with advice on policy design to better achieve the overall Sustainable Development Goals. As the Chinese government is promoting waste sorting to solve the garbage problems throughout the country, this paper is instructive for local governments to design and evaluate their policies to optimise the overall goal of reducing waste from the upstream consumption behaviours and the downstream waste-sorting ones.

## 2. Literature Review

### 2.1. Pro-Environmental Behaviour and the Spillover Effect

In terms of the meaning and classification of pro-environmental behaviours and spillover effects, Steg and Vlek [14] believe that PEBs minimise environmental damage and promote behaviours that are beneficial to the environment. Two types of definitions of PEB spillover effects exist. One is that past PEBs affect the possibility or the extent to which people participate in other PEBs, and the other refers to the strengthened or weakened effects of regulatory policy on non-targeted behaviours [3,4]. The influence direction of spillover effects is divided into positive and negative spillover effects. A positive spillover effect refers to the occurrence of one environmentally friendly behaviour that drives other environmentally friendly behaviours [15]. By contrast, a negative spillover effect refers to the occurrence of an environmentally friendly behaviour that inhibits other environmentally friendly behaviours [7].

The empirical research on the spillover effects of PEB mostly focuses on initial and subsequent behaviours [16]. Most of the relevant studies have confirmed the existence of light to moderate spillover effects. Among these works, Thomas et al. [17] found that Wales’s one-time shopping bag charging policy promotes consumer reuse of shopping bags as well as encourages relevant sustainable behaviour fulfilment. Xu et al. [10] found positive spillover effects from incentive policies in a study on the classification of household waste in Hangzhou. Although these two investigations attempted to explore external interventions’ influences on spillover effects, they have obvious shortcomings in that the effects of different kinds of policies have not yet been separated and compared in these studies. The purchase of environmentally friendly products is found to be positively related to the support for wind power development [18,19]. However, Truelove and Nugent. Ref. [20] found the opposite result by reporting that reducing plastic straw use does not lead to changes in policy support.

In terms of research objects, previous research mainly focuses on initial and subsequent behaviours to explore the spillover between them, as well as the mediators in this process. Some studies also seek to explore different interventions, such as nudges and the information framing’s impact on spillover [21,22,23]. However, few studies focus on public policy’s spillover effects, let alone conduct a comparison between different kinds of policies. Prior studies have also verified that certain regional differences exist in pro-environmental behaviours [16]. This outcome indicates that spillover effects may show different patterns in different areas. Considering the aforementioned factors, this work investigates the waste-sorting behaviour and the relevant spillover effect onto sustainable consumption in a Chinese policy context.

### 2.2. Regulatory Policy and the Spillover Effect

As defined by Truelove et al. [9], pro-environmental interventions include any attempt to encourage behaviour change, such as ‘a request to perform a new behaviour, public education campaign, tax incentive, provision of green infrastructure such as kerbside recycling, and regulatory policy’. For the classification of regulatory policy, Messik et al. [24] and Wang et al. [25] divided PEB regulatory policies into informational and structural strategies. Yang et al. [26] classified pro-environmental policies to promote EVs into three categories: information policy, subsidy and convenience policy (2019). Mi [27] categorised Chinese environmental policies into command and control policy, financial incentives, information policy and voluntary participation policy.

However, scarce research exists on the spillover effects of different regulatory policies under a real policy context. For instance, Thomas et al. [17] examined the spillover effects of the Welsh Single-Use Carrier Bag Charge policy, which found that the increased use of one’s bags is linked to increases in six other PEBs. However, how different intervention policies lead to different spillovers has not been well studied. As another type of intervention defined by Truelove et al. [9], information framings have been explored in experiments to test the spillover effect. Monetary framing can prohibit positive spillovers or even lead to negative ones [28,29]. Certain studies indicate that the type of information framing for PEB may be one important factor that classifies different spillover effects [28,30,31]. For example, Steinhorst et al. [29] used a field experiment to highlight the negative side effects of monetary framing in the context of PEB and found that the positive spillover on climate-friendly intentions manifests in the environmental framing condition only. No effect was found for monetary framing. Previous studies have demonstrated that one PEB can activate people’s intentions for other PEBs by rousing the underlying pro-environmental values or environmental goals.

To solve the problem of accelerated increases in the generated waste quantities, the Chinese government launched the Mandatory Waste Sorting Program (Draft) about 5 years ago in 2016. As one of the first pilot cities of waste sorting, the Shanghai municipal government first established a regulation on the administration of domestic waste in 2019. Four categories of waste were defined on the residential side, namely recyclable, hazardous, wet and dry waste [32]. Different kinds of waste must be thrown into correspondent bins. To encourage residents to follow the waste management rules, a 50–200 yuan penalty was implemented to punish those who dispose of their waste in an irresponsible and illegal manner. In the early stage, propaganda and education on waste sorting through various ways were conducted. Online ways, such as online news media and social media such as Weibo and WeChat, and offline ways, such as TVs and newspapers, were widely used in the propaganda of the waste-sorting policy, aiming at letting people understand the importance of waste sorting. In some communities, only basic information and knowledge were provided to the residents as guidelines for their behaviours. Whether the residents abide by the regulatory policy is based on their own decisions, which indicates that the policy is not compulsive. This policy context was defined as ‘voluntary participation’, which was one of the pro-environmental policy types according to Mi [27]. For those communities with enough human capital to work as volunteers, people who did not throw waste correctly and refused to abide by the policy would be fined. Both the policies are implemented by the community. Whether a neighbourhood chooses the voluntary participation policy or the penalty policy chiefly depends on the administrative capability and whether enough volunteers can be recruited to supervise the waste-sorting process. Thus, no evidence links policy implementation with the characteristics and preferences of the residents in different neighbourhoods.

The penalty policy, as a negative way to influence the private benefits of people, may reinforce the undesirable mindset that pro-environmental behaviour is only deemed worthwhile when personal side financial benefits are involved [33]. Thus, based on this logic, it is reasonable to infer that the penalty policy may decrease people’s intrinsic motivation to conduct PEBs unless external requirements are added. By contrast, the voluntary participation policy, which keeps the initiative in people’s own hands, will arouse their internal motivations to sort waste and is likely to lead to a positive spillover. Although some communities in Shanghai provide ‘points’ for redeeming rewards as an incentive for residents to sort waste, the standards and forms of the rewards vary considerably in different neighbourhoods, a feature that may lead to divergent effects on people’s attitudes and behaviours. Therefore, this kind of policy is excluded from this study. Hence, we formulated the following hypothesis:

**Hypothesis** **(H1).** *The voluntary participation policy targeted at waste-sorting behaviour increases people’s non-targeted sustainable consumption behaviour (beyond the realm of waste sorting). The penalty policy targeting at waste sorting behaviour decreases people’s non-targeted sustainable consumption behaviour.*

### 2.3. Psychological Mechanism of the Spillover Effect

Scholars in this field proposed a series of related theories on the influencing factors of pro-environmental and sustainable consumption behaviours. Some theoretical and empirical studies explored the influencing factors of pro-environmental behaviour spillover effects, but few systematic investigations were conducted on the formation mechanism of spillover effects. The existing research on the influencing factors of spillover effects focuses on the related theoretical basis and the intermediary role of several psychological factors.

Scholars have examined the impact of various factors, such as values, attitudes, norms, knowledge, cultures, motivations and behavioural intentions, on pro-environmental and sustainable behaviours [25,26,34,35]. Through a questionnaire survey, Wang et al. [25] found that consumers’ attitudes, subjective norms and perceived behaviour control significantly affects the willingness to purchase new energy vehicles. Various studies have also highlighted the identity–behaviour link [29,36]. For example, people who see themselves as typical recyclers are more likely to recycle than those who do not perceive themselves as recyclers [37]. Environmental self-identity influences people’s pro-environmental behaviour because it elicits feelings of moral obligation and the willingness to coincide with their notions of self. Further work demonstrates that environmental identity mediates positive PEB spillover effects. Informing people about the positive effects of their pro-environmental behaviours would make them perceive themselves as the type of persons who are concerned about the environment. This would help them build a pro-environmental identity, which would then guide their behaviours to engage in more PEBs [31]. For example, Peters et al. [36] verified that individuals with stronger environmental self-identity are also likely to participate in other sustainable energy consumption behaviours. Although the mediating effect of environmental self-identity in a positive spillover has been demonstrated by the aforementioned studies, whether it can also mediate a negative spillover remains unexplored. As revealed by Truelove et al. [9], monetary framing tends to reduce people’s inner motivation to conduct pro-environmental behaviour, as it places external pressure on them. Hence, it is reasonable to assume that the initial behaviours stimulated by external pressures tend to lower the people’s obligation motivation to conduct PEB, a feature that is strongly associated with self-identity. Such a regulatory policy may eventually reduce people’s environmental self-identity and lead to a negative influence on other PEBs. Hence, we formulated the following hypothesis:

**Hypothesis** **(H2).** *The effect of the regulatory policy targeted at waste sorting on sustainable consumption behaviour is mediated by environmental self-identity. Environmental self-identity positively mediates the relationship between voluntary participation policy and sustainable consumption behaviour. Environmental self-identity negatively mediates the relationship between penalty policy and sustainable consumption behaviour.*

As stated in Lacasse [38], people may feel guilty if they perceive that they are failing to perform PEBs. Kantola et al. [39] and Osbaldiston and Schott [40] indicated that being reminded of past environmentally harmful behaviours can lead people to perform PEBs. Although certain studies believe that guilt may be alleviated once a PEB is performed and that motivation to perform additional pro-environmental behaviours can be reduced, no coinciding empirical results have been found on this point. As deducted by this study, the external stimulus may be a crucial condition that influences the change of guilt. When the initial behaviour is triggered by an external intervention, the change of guilt may be more ambiguous. If the external trigger is environmental, then policies would aim at arousing people’s inner willingness and telling them what they can do for the environment, an endeavour that may make people realise that they could have done more for the environment. Thus, people’s guilt may increase under a non-monetary voluntary participation policy. Hence, we formulated the following hypothesis:

**Hypothesis** **(H3).** *The effect of the regulatory policy targeted at waste sorting on sustainable consumption behaviour is mediated by guilt. Guilt positively mediates the relationship between voluntary participation policy and sustainable consumption behaviour. Guilt negatively mediates the relationship between penalty policy and sustainable consumption behaviour.*

## 3. Methods

### 3.1. Study Area

The study was conducted in Shanghai and Beijing, with Shanghai citizens in the regulatory policy group and Beijing citizens in the control group. Shanghai was chosen because it was one of the first batch of waste-sorting pilot cities. On the contrary, Beijing was not included in the waste-sorting pilot cities during the time frame of this study, which had not begun implementing the waste-sorting policy until 1 May 2020. Although some communities of Beijing had implemented waste sorting for years, the residents of those limited communities were excluded from the sample in this study. Thus, Beijing was selected as the control group to help us verify the spillover effects of regulatory policies implemented in Shanghai. These two cities were compared because the residents’ socio-demographic characters are similar.

### 3.2. Measures

On the basis of our hypotheses and the literature review, we considered the regulatory policy as an independent variable and sustainable consumption behaviours as dependent variables, respectively. Environmental identity and guilt were the mediators between this relationship. An online questionnaire, which comprises four parts, was developed. In this study, the analysis was mainly based on the data from Parts 2 (regulatory policy and psychological factors), 3 (sustainable consumption behaviour) and 4 (socio-demographic factors). Except for the socio-demographics, all other items are measured with the five-point Likert scale. The majority of the measures are based on the validated scales from previous studies, and we further modified a number of items to satisfy the context of this research. All the measurement items can be read in Table A1 in the Appendix A.

The regulatory policy is measured through the following question: What type of waste-sorting policy does your community implement? The participants can choose among options of ‘penalty policy’, ‘voluntary participation policy’, ‘both’ and ‘none’. The community is the grassroots enforcement unit of the waste-sorting policy, and different communities use different policies to promote waste sorting. For example, some communities where conditions permit imposing a fine on those who do not sort waste properly under the supervision of volunteers, while some provide knowledge and facilities of waste sorting to their residents but whether waste sorting is performed or is not is totally voluntary. Only participants from Shanghai were asked to answer this question because Beijing had not implemented policies on waste sorting yet during the study period and was a control group here.

The environmental self-identity is measured by adapting three items from van der Werff et al. [41]. Respondents are asked to rate them using a five-point scale ranging from 1 (‘Strongly disagree’) to 5 (‘Strongly agree’). The sample items include ‘Acting environmentally friendly is an important part of who I am’. Two items are used to measure guilt, which are adapted from Harth’s research [42]. Respondents are asked to rate them using a five-point scale ranging from 1 (‘Strongly disagree’) to 5 (‘Strongly agree’). The sample items include ‘I feel guilty about my behaviour, which is relevant to the environment’. The corresponding Cronbach’s α for these variables is 0.768 and 0.865, respectively.

Sustainable consumption behaviour is measured in the questionnaire through four items adapted from Lacasse’s (2016) research. Respondents are asked to rate them using a five-point scale ranging from 1 (‘Never’) to 5 (‘Very often’). The sample items include ‘I purposefully look to buy products with less packaging’. The scales are reliable, with Cronbach’s α = 0.805.

### 3.3. Survey Deployment

We designed a survey questionnaire to be distributed in Shanghai in order to get responses towards the waste management regulatory policy and sustainable consumption behaviour. As the counterpart, the Beijing residents’ responses were collected without asking questions about the regulatory policy. We chose these two cities by considering the fact that the residents’ education level, income level and relevant socio-demographics are similar. The data were collected online through a professional survey company during the time period of October to November 2019. In total, 500 questionnaires were distributed, with 300 from Shanghai and 200 from Beijing. By deleting those questionnaires that failed the attention test, we received 360 valid questionnaires, which yielding a valid response rate of 72%.

### 3.4. Analysis Strategies

Descriptive statistical analysis was first conducted to analyse the general trends in behaviours. To compare the voluntary participation policy’s and the penalty policy’s effects, individuals who chose mixed policies (‘both’) and ‘none’ for the regulatory policy variable were selected for analysis. Respondents who chose ‘voluntary participation policy’ were assigned a value of 1 for the regulatory policy variable, while those who chose ‘penalty policy’ were assigned a value of −1 for this variable. Secondly, the sample of Beijing was merged with the sample of Shanghai to act as the control group so as to describe the baseline. Correspondingly, the regulatory policy variable of participants from Beijing was assigned a value of 0 so that the policy’s effect could be analysed by a one-time mediation test. Such a method was also used by Thomas et al. [17] in comparing the policy’s impact. Although the samples of Shanghai and Beijing resembled each other in many ways, the difference in income distribution might have led to an underestimation of the baseline, which is further discussed in Section 4.1. A mediation effect examination was conducted through a bootstrap test (PROCESS Model 4) [43]. Specifically, model 4 (a mediation model) in Hayes [43] and the PROCESS macro in SPSS 23.0 were used to test Hypotheses 1–3.

Bootstrapping was applied here since it is a method that resamples from an original sample to derive a more accurate estimate than is found through traditional methods. Both direct and indirect effects of the conceptual model were tested using the SPSS PROCESS macro. Hayes [43] stated that this approach allows estimation of both indirect and interaction effects using bootstrapping procedures based on generating multiple random samples. As raised by Dermody et al. [44], ‘Simulation studies confirm that bootstrapping is more powerful than the original Baron and Kenny [45] method of testing mediation by using the causal steps approach and has several advantages over the Sobel test [46,47].’ The bootstrap not only provides stronger accuracy in confidence intervals [48] but also tests a model’s predictive validity, which has led to it receiving increasing attention in recent years [49]. This approach is widely used in behavioural studies in various areas, including pro-environmental behaviours and consumption behaviours [50,51].

## 4. Results

### 4.1. Sample Characteristics and Tests for Constructs

The demographic profile indicated that the survey respondents were mostly young and middle aged and well educated (see Table 1). Among the Shanghai and Beijing respondents, 57.5% and 47.5% were in the age range of 18–30 years and 26.3% and 28.3% were in the age range of 31–40 years, respectively. For the education level, those holding a junior college or college degree accounted for 62.9% and 52.7% of the Shanghai and Beijing respondents, respectively, while 26.3% and 35.8% of the respondents in Shanghai and Beijing held a graduate degree, respectively. This implies that there is a bias related to the characteristics of age and education. This limitation may have arisen from the fact that potential participants were recruited online. Among the Shanghai and Beijing respondents, 45.0% and 45.8% were male, which was slightly lower than the percentage of men in Chinese urban areas in 2019 (51.1%). The sample covered the entire range of income levels. Those with an annual household income after tax of less than 100,000 CNY accounted for 44.2% of the Shanghai sample and 25.8% of the Beijing sample, and those with an annual income between 100,000 and 150,000 CNY accounted for 34.2% and 37.5% of the Shanghai and Beijing samples, respectively. Chi-tests were conducted through SPSS 21.0 to examine whether the samples from Shanghai and Beijing are similar. The results showed that all of the sample characteristics of these two cities do not exhibit a significant difference except for household income. The income distribution of Beijing and that of Shanghai were found to be statistically significantly different from each other, where the population of Beijing had a relatively higher income. Since a higher income level has been found to correlate with lower environmental concern and behaviour in previous studies as well as this one under a Chinese context, this bias may lead to a slight underestimation of the baseline described by Beijing respondents. The impact of the bias will be further discussed in the Discussion section. For data details, please see Table 2.

The loadings, composite reliability, the average variance extracted (AVE) and Cronbach’s α are shown in Table 2. It can be seen that the factor load of each item was greater than 0.5, indicating that each item had good internal consistency. Cronbach’s α, CR and AVE of each variable also reached acceptable levels. The results show that the scale used in this study has good reliability, convergent validity and discriminant validity.

### 4.2. Descriptive Statistical Analysis

Table 3 shows the means, standard deviations and correlation coefficients of all variables. It can be seen that regulatory policy and environmental self-identity were positively correlated, as were guilt and sustainable consumption behaviour (r = 0.141, *p* < 0.05; r = 0.177, *p* < 0.01; r = 0.157, *p* < 0.05). Environmental self-identity was positively correlated with guilt (r = 0.462, *p* < 0.001) and sustainable consumption behaviour (r = 0.500, *p* < 0.001), and guilt was positively correlated with sustainable consumption behaviour (r = 0.417, *p* < 0.001).

**Figure 1:**
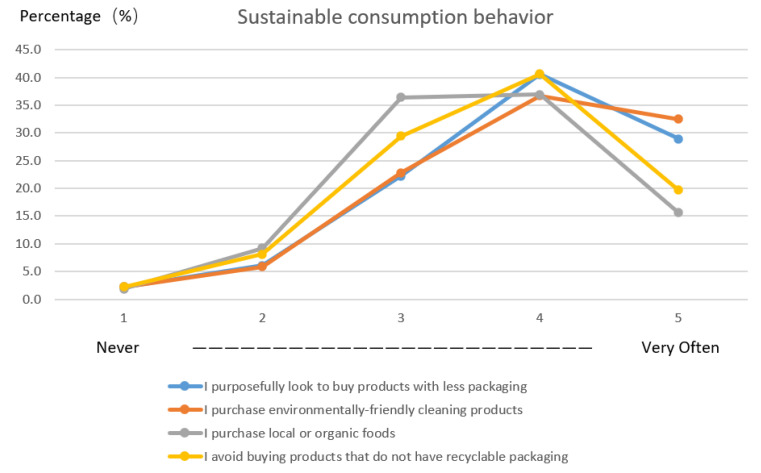
Frequency of response to sustainable consumption behaviour.

The frequency data in Figure 1 show that among the four sustainable consumption behaviours, people purposefully purchase products with less packaging and choose environmentally friendly cleaning products more frequently. Nearly 70% of the participants responded that they ‘very often’ and ‘often’ perform these two behaviours. By comparison, the percentage of participants who stated that they ‘very often’ or ‘often’ purchase local or organic food was slightly lower (52.5%).

### 4.3. Tests of Hypotheses

The regulatory policy had a significant and positive impact on sustainable consumption behaviour (β = 0.164, *p* < 0.05). Therefore, Hypothesis 1 was supported. We explored the mediation effect through the bootstrap test (PROCESS model 4) [43]. As shown in Table 4, the regulatory policy had significant and positive effects on environmental self-identity (β = 0.460, *p* < 0.001). Respondents who are driven by the voluntary policy generated significantly higher levels of environmental self-identity than those in the other two groups. By contrast, the levels of environmental self-identity of respondents who are driven by the penalty policy were significantly lower than those in the other two groups. Similarly, the regulatory policy had significant and positive effects on guilt (β = 0.218, *p* < 0.01). Respondents who are driven by the voluntary participation policy had significantly higher levels of guilt than those in the control and penalty groups, while those who are driven by the penalty policy had significantly lower levels of guilt than those in the control and voluntary participation groups. The regulatory policy’s effect on sustainable consumption behaviour became insignificant in model 3 (β = 0.065, *p* > 0.05), an outcome which indicates that this effect is wholly mediated by environmental self-identity and guilt. Moreover, environmental self-identity and guilt also had significant positive impacts on sustainable consumption behaviour (β = 0.460, *p* < 0.001; β = 0.194, *p* < 0.001).

**Figure 2:**
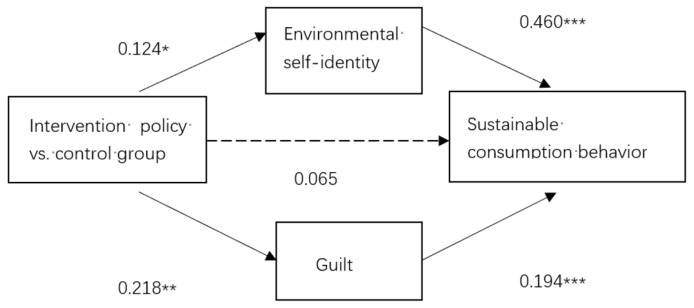
Influence of regulatory policy on sustainable consumption behaviour through the mediation of environmental self-identity and guilt. *** *p* < 0.001, ** *p* < 0.01, * *p* < 0.05. The assignment for regulatory policy: penalty policy = −1; control = 0; voluntary participation policy = 1. A continuous line specifies a significant relationship. A dotted line indicates that the relationship is not significant at the 5% level (one-sided for β-coefficients).

As can be seen from Table 5 and Figure 2, the analysis of the mediating effects of environmental self-identity and guilt between regulatory policy and sustainable consumption behaviour shows that the indirect effect of environmental self-identity on regulatory policy to sustainable consumption behaviour was 0.057, while its bootstrap 95% confidence interval did not include 0, thereby indicating a significant mediating effect of environmental self-identity between regulatory policy and sustainable consumption. The indirect effect of guilt on regulatory policy to sustainable consumption behaviour was 0.042, and its bootstrap 95% confidence interval did not contain 0, an outcome that also showed a significant mediating effect of guilt on regulatory policy and sustainable consumption. These results supported Hypotheses 2 and 3.

## 5. Discussion

### 5.1. Result Discussion

The analysis revealed that waste-sorting policies can significantly influence people’s upstream sustainable consumption behaviours, while different regulatory policy types produce either an increase or a decrease in people’s sustainable behaviour. With regard to behavioural spillover, the voluntary participation policy was anticipated to induce a positive spillover effect on sustainable consumption behaviour. For the penalty policy group, a negative spillover effect was expected. Comparisons between different regulatory policy groups and control groups confirmed these assumptions. The positive spillover of the voluntary participation policy can be explained by the fact that what people choose to do is in accordance with their intrinsic intentions, rather than an outside stimulus such as penalties. This situation leads to an increase in people’s PEB-relevant psychological factors, which, in turn, can arouse people’s inner motivation to engage in subsequent PEBs. This study also demonstrated that a policy providing monetary punishment is likely to induce a negative spillover effect on other PEBs. The role of penalty policy has never been examined in previous spillover research. We can assume that people who are driven by this kind of policy are likely to view PEB as something irrelevant to themselves, and they only engage in this behaviour under external pressure, rather than because of intrinsic motivation. The self-motivated behaviour is transformed into externally driven behaviour, a circumstance that may reduce people’s inner willingness to engage in similar behaviours (specifically sustainable consumption behaviour in this research). At the same time, the bias caused by the sample differences between Shanghai and Beijing should be considered. Given that the respondents from Beijing have relatively higher incomes than those from Shanghai, this divergence may lead to an underestimation of the baseline described by Beijing respondents. That is, the negative effect of the penalty policy is slightly underestimated, but the positive effect of the voluntary policy is slightly overestimated. On the basis of the relationship between different regulatory policies and spillover effects, we can conclude that a regulatory policy should be precisely designed because different types of policy may even show inverse effects on subsequent PEBs. Therefore, the regulatory policy effect should be evaluated from the perspective of its overall environmental influences. In this case, the aims of solving the problem of ‘garbage sieges’ and realising a more sustainable society are driven not only by people’s waste-sorting behaviours but also by their upstream sustainable consumptions. Priority should be accorded to a policy that can promote more relevant behaviours. More importantly, considering the fact that changing people’s consumption patterns has long been a difficult problem [52], contributing to waste sorting may lead to unexpected effects.

The results also add to our knowledge of possible spillover mechanisms and intervention paths. Our results revealed that positive and negative spillovers are completely mediated by environmental self-identity and guilt for the environment. This means that both environmental self-identity and guilt show double effects in either a positive or a negative spillover. However, previous research found that self-identity mainly mediates a positive spillover but guilt only mediates a negative spillover [9,38]. A possible explanation for their double effect in this research is as follows: A penalty policy would lead people to believe that they conduct PEBs to avoid punishment and monetary loss, a notion that may weaken their perceived role as environmentalists. Moreover, a voluntary participation policy reinforces the people’s guilt for the environment through showing people that they can do more for the environment than they have done previously, thereby possibly leading people to think they have done excessively little for the environment. This perception may increase their guilt for the environment. Understanding the double effects of self-identity and guilt helps us better intervene through mediators to increase the positive spillover or decrease the negative spillover.

This study contributes to the existing literature in several ways and provides a foundation for future research. Firstly, sustainable consumption is a vital way to help minimise the use of natural resources, toxic material usage and emissions of waste and pollutants so as to reach Sustainable Development Goals. Previous research has focused on the psychological and contextual factors that would affect such behaviours but has seldom considered the influences that arise from other behaviours or policies. This work provided us with a new perspective for understanding sustainable behaviours and their correlations with relevant behaviours and policies and thus provides academics and practitioners with a new perspective for promoting the transition of sustainable consumption. Secondly, our work contributes to the literature on the spillover effect [9,29] by showing that different regulatory policies show different spillovers on subsequence PEBs. Note that the penalty policy for waste-sorting behaviour can spill over into consumption and lead to a decrease in sustainable consumption behaviour. Conversely, the voluntary participation policy leads to a significant increase in people’s sustainable consumption behaviour. The results contradicted the widespread approaches of environmental campaigning, which assumed that people will be motivated to behave in an environmentally friendly way after performing a PEB [7]. This current research shows that this spillover effect can only motivate subsequent PEB under a voluntary participation policy. The negative spillover effect found under a penalty policy indicates that carefully choosing the motives upon which the appeal of environmental campaigns is directed [53] is essential. Thirdly, this study was conducted under a real policy context, a feature that is lacking in the previous literature based on laboratory design [5]. The result of this work was drawn according to a comparison of the respondents with people in areas where no waste-sorting policy is in place. This design makes the outcomes more reliable than those of some previous studies without a control group. Fourthly, the evaluation of the spillover effects of waste-sorting policies through combining a targeted behaviour and a non-targeted behaviour has a highly practical value and provides policymakers with suggestions about which policy should be strengthened and modified to reach the overall Sustainable Development Goals. Few researchers have paid attention to the possible spillover effect on people’s upstream consumption patterns when evaluating waste-sorting policies. As the Chinese government is promoting waste-sorting behaviour throughout the country, the result of this work is of great significance for local governments in designing and evaluating their policies to better achieve the overall goal of reducing and better managing waste, from the perspectives of upstream consumption behaviours and downstream waste-sorting behaviours. 

### 5.2. Managerial Implications

We argued that the waste-sorting policy of Shanghai, although effective at promoting waste sorting by residents, is not always as effective at encouraging wider behavioural changes when external pressure is applied. As the goal of a waste-sorting policy is to reduce the amount of garbage in the long run, a sustainable consumption pattern is also an important aspect to realising this goal. However, when a penalty policy is launched, people tend to behave unsustainably in their daily consumption, although they may have high levels of waste-sorting behaviours, and this reaction may increase the garbage they produce. By contrast, a voluntary participation policy contributes to the increase in people’s environmental self-identity and guilt for the environment, thereby leading to more sustainable consumption. At present, more Chinese cities are introducing waste-sorting policies. Policymakers should take full account of the comprehensive impact of policies on the environment and choose policies carefully. Penalty policies should be replaced by policies that can trigger the people’s inner motivation to transform negative into positive spillovers. For those cities that have already launched those policies, the psychological process may be a point of penetration for intervention. Governments should raise the residents’ environmental self-identity and guilt for the environment through various forms of publicity and education to realise the overall goal of reducing garbage.

As inferred from the conclusion of this study, different policies that target certain pro-environmental behaviours lead to different spillover effects on other behaviours. Our results emphasise the risks of appealing to external pressures or self-interest when promoting environmental behaviour. Providing sufficient knowledge and information and safeguarding the infrastructure needed to conduct the behaviour ought to be the preferred strategies for promoting behavioural change for environmental protection. More voluntary participation policies and policies that can stimulate intrinsic motivations should be implemented to increase the overall behaviours towards a sustainable society. Penalty policies and policies that can apply external pressure should be carefully considered before being introduced so as to prevent negative spillover effects on other sustainable behaviours and negative influences on the environment in the long run. Generally, this work sheds light on the fact that policymakers should consider the direct effects of policies and the corresponding spillover effects on other non-targeted pro-environmental behaviours. The government should formulate policies on the basis of the overall environmental effects of those policies.

## 6. Conclusions

The aim of this study was to verify whether people’s sustainable consumption behaviours can be influenced differently by waste-sorting policies from the perspective of spillover. Specifically, it compared the effects of voluntary participation and penalty regulatory policies for waste sorting with regard to their spillover effects on sustainable consumption behaviour (non-targeted behaviour) beyond the realm of waste sorting, thereby demonstrating that different regulatory policies of waste sorting do change people’s consumption patterns differently.

Results show that a penalty policy significantly decreases people’s sustainable consumption behaviours through a negative spillover effect, but a voluntary participation policy significantly increases people’s sustainable consumption behaviours through a positive spillover effect. Environmental self-identity and guilt were found mediated not only positive but also negative spillover effects. This indicates that a waste-sorting policy should be carefully designed because different policy instruments may lead to different environmental influences. Compared to a penalty policy, a voluntary policy may be more propriate because it can arouse a positive spillover effect on other environmental behaviours such as sustainable consumption. Both sustainable consumption and waste sorting contribute to the decrease in waste amount and relevant pollution. A penalty policy, which imposes restrictions on people’s waste-sorting behaviour, will decrease people’s sustainable consumption willingness, which means even worse performance in other pro-environmental fields. Besides, the mediation effects of environmental self-identity and guilt demonstrated in this study also provide important information. For those cities where a penalty policy has already come into effect, the policymakers and relevant organisations can also try to decrease the policy’s negative effect through raising people’s environmental self-identity or their guilt for their environmentally unfriendly behaviours.

This study fills the gap and contributes to the current spillover effect studies in the pro-environmental behaviour realm from the perspective of regulatory policies. The results herein can be used to provide implications for policymaking in waste management and other pro-environmental fields to achieve the overall Sustainable Development Goals through the promotion of the transition of multiple behaviours.

This study has several limitations that should be emphasised in future research. Firstly, more psychological factors other than environmental self-identity and guilt may mediate the spillover effect between regulatory policies and secondary behaviours. Future studies can try to explore more mediation factors’ effects on spillovers. Secondly, this research sheds light on the wide range of consequences that different types of policies can have on sustainable consumption behaviours in the short term. The time range between the launch of a policy and its spillover effect is approximately 4–5 months. As a previous study revealed, the spillover effect of policies on non-targeted behaviours weakens over time [17]. This study did not trace long-term behavioural change, so it remains unclear whether positive and negative spillovers decrease with time. Future studies can design and trace the behavioural change over a longer period. Thirdly, people’s knowledge about waste sorting is different, which was not considered in this study. Knowing how waste will be dealt with after sorting may influence people’s willingness to sort the waste. For example, if people believe that most of the waste will be burned and used to provide a supplementary source of heat and energy, they may sort less. On the contrary, if there would be combinations of various waste management strategies, such as composting, landfilling, anaerobic digestion, incineration and recycling, people may have higher intentions to engage in waste sorting. At the moment, Chinese people’s knowledge about waste usage is still limited. In future, research can try to investigate whether informing people about specific usages would help increase their participating willingness in waste sorting.

## Figures and Tables

**Table 1:** Sample characteristics.

Category		Shanghai (*n* = 240)		Beijing (*n* = 120)	
		Frequency	Percentage (%)	Frequency	Percentage (%)
Gender	1. Male	108	45.0	55	45.8
	2. Female	132	55.0	65	54.2
Age (years)	1. ≤18	9	3.8	2	1.7
	2. 18–30	138	57.5	57	47.5
	3. 31–40	63	26.3	34	28.3
	4. 41–50	29	12.1	22	18.3
	5. Over 50	1	0.4	2	1.7
Education	1. Senior middle school or lower	26	10.8	9	7.5
	2. Junior college or college degree	151	62.9	68	56.7
	3. Graduate degree	63	26.3	43	35.8
Household income	1. Under ¥100,000	106	44.2	31	25.8
	2. ¥100,000–150,000	82	34.2	45	37.5
	3. ¥150,000–250,000	23	9.6	17	14.2
	4. ¥250,000–350,000	16	6.7	17	14.2
	4. Over ¥350,000	13	5.4	10	8.3

Notes: *n* = 360.

**Table 2:** Loading, composite reliability and the average variance extracted.

Items	Loading	Composite Reliability	Average Variance Extracted	Cronbach’s α
ESI1	0.839	0.875	0.700	0.768
ESI2	0.861			
ESI3	0.809			
GFE1	0.939	0.937	0.882	0.865
GFE2	0.939			
SCB1	0.755	0.873	0.632	0.805
SCB2	0.790			
SCB3	0.774			
SCB4	0.857			

ESI: environmental self-identity; GFE: guilt for the environment; SCB: sustainable consumption behaviour.

**Table 3:** Means, standard deviations and correlations.

Variables	Mean	Standard Deviations	1	2	3	4
1. Regulatory policy	0.01	0.53	1			
2. Environmental self-identity	4.05	0.41	0.141 *	1		
3. Guilt	3.91	0.81	0.177 **	0.462 ***	1	
4. Sustainable consumption behaviour	3.67	0.58	0.157 *	0.500 ***	0.417 ***	1

Notes: *n* = 237. *** *p* < 0.001, ** *p* < 0.01, * *p* < 0.05. Regulatory policy is coded as −1 = penalty policy (Shanghai), 0 = control without policy (Beijing) and 1 = voluntary participation policy (Shanghai).

**Table 4:** Results for mediating effects.

	Model 1 Environmental Self-Identity (M1)			Model 2 Guilt (M2)			Model 3: Sustainable Consumption Behaviour (Y)			Model 4: Sustainable Consumption Behaviour (Y)		
	Coefficient	SE	t	Coefficient	SE	t	Coefficient	SE	t	Coefficient	SE	t
Constant	4.043	0.0412	98.043 ***	3.907	0.058	67.891 ***	1.048	0.280	3.738 ***	3.664	0.049	74.692 ***
Regulatory policy(X)	0.124	0.057	2.189 *	0.218	0.079	2.755 **	0.065	0.059	1.103	0.164	0.068	2.429 *
Environmental self-identity (M1)							0.460	0.074	6.215 ***			
Guilt (M2)							0.194	0.053	3.655 ***			
R^2^	0.020			0.031			0.298			0.025		
F	4.790 *			7.587 **			32.901 ***			5.902 *		

Notes: *n* = 237. *** *p* < 0.001, ** *p* < 0.01, * *p* < 0.05.

**Table 5:** Indirect effect of regulatory policy on sustainable consumption behaviour through environmental self-identity and guilt.

	Effect	SE	95% CI
Total indirect effect	0.099	0.036	[0.029, 0.174]
Environmental self-identity	0.057	0.026	[0.009, 0.110]
Guilt	0.042	0.022	[0.008, 0.094]

Note. Bootstrap resample = 5000. SE: standard error; CI: confidence interval. Estimates were calculated using the PROCESS macro.

## Data Availability

The data required to reproduce these findings cannot be shared at this time as the data also forms part of an ongoing study.

## Appendix A

**Table A1:** Description of measurement items.

Variables	Measurement Items
Regulatory policy	What type of waste-sorting policy does your community implement?
Environmental self-identity	Acting environmentally friendly is an important part of who I am.
	I am the type of person who acts environmentally friendly.
	I see myself as an environmentally friendly person.
Guilt	I feel guilty about my behaviour relevant to the environment.
	I am regretful about my behaviour relevant to the environment.
Sustainable consumption behaviour	I purposefully look to buy products with less packaging.
	I purchase environmentally friendly cleaning products.
	I purchase local or organic foods.
	I avoid buying products that do not have recyclable packaging.
